# Physical medicine and rehabilitation clinical experiences: A narrative review of curricula and educational interventions

**DOI:** 10.1002/pmrj.13262

**Published:** 2024-09-13

**Authors:** Cara Vernacchia, Elizabeth Brown, Priya Mhatre, Leslie Rydberg

**Affiliations:** ^1^ Department of Physical Medicine & Rehabilitation Northwestern University Feinberg School of Medicine Chicago Illinois USA

## Abstract

Physical medicine and rehabilitation (PM&R) is a specialty of medicine that focuses on function and the care of people with disabilities. Many medical schools offer PM&R content by means of PM&R clerkships for career development purposes with varying curricula and assessments; however, there is limited information regarding the optimal way to teach clinical skills relating to the field of PM&R. This narrative review study was performed to evaluate PM&R specific clinical curricular interventions. The review included a PubMed search that yielded 63 articles and a Cumulated Index to Nursing and Allied Health Literature (CINAHL) search of 175 articles. A total of 14 articles were selected for review. PM&R clerkships were found to have a variety of educational interventions, including inpatient and outpatient clinical experiences, lectures, Objective Structured Clinical Examinations (OSCEs), case discussions, written examinations, physical examination sessions, cadaver sessions, small group discussions, and virtual education. PM&R rotations can improve neurologic and musculoskeletal physical examination skills, PM&R specific knowledge, and confidence in PM&R skills. More research is needed to determine the optimal methods to teach and assess PM&R knowledge and skills in the clinical setting to drive future PM&R curriculum development and educational innovations.

## INTRODUCTION

Physical medicine and rehabilitation (PM&R) physicians, also known as physiatrists, treat a wide variety of medical conditions affecting the brain, spinal cord, peripheral nerves, and musculoskeletal system. The American Academy of Physical Medicine and Rehabilitation^1^ states that PM&R physicians diagnose and treat pain and dysfunction due to an injury, illness, or disabling condition and focus management on function with a specific expertise in working with patients with neurologic, musculoskeletal, and chronic medical conditions. They lead and collaborate with interdisciplinary teams to optimize patient care and treat the whole person. Physiatrists are also practiced in other important skills that are considered desired outcomes of undergraduate medical education regardless of chosen specialty, such as patient‐centered medical care, effective communication and interpersonal skills, system awareness, and team‐based care.^1^ PM&R physicians may also be involved in medical education relating to the care of people with disabilities.^2^, ^3^

Students may seek clinical experiences in PM&R such as clerkships or externships for career development purposes. However, because PM&R is not one of the required core third‐year clerkships and many medical schools are not affiliated with a PM&R residency program or department, not every medical student in the United States will have exposure to PM&R clinical experiences or curriculum. A study by Benbassat et al. in 2021 found that 64% of medical schools had an elective PM&R clerkship option, and only 2.5% had a required PM&R clerkship.^4^ Required clerkships were typically 2 weeks long, and elective clerkships were usually 4 weeks in duration. As PM&R is rarely a required clerkship, physiatrists may be less involved than other specialties in medical school curriculum planning, and there are few data available on the actual educational practices and curricula that are being used both within and outside of these PM&R clerkships.

There are no published consensus or guidelines on the optimal way to teach clinical skills relating to the field of PM&R. Other medical specialties have started to review published medical literature to determine how to optimize teaching through evidence‐based education.^5^, ^6^, ^7^ As such, the purpose of this narrative review was to scrutinize the published literature to examine the methods, settings, and programs in which PM&R clinical skills are taught and assessed, in order to inform future curriculum development and innovation for students seeking PM&R experiences.

## METHODS

**FIGURE 1:**
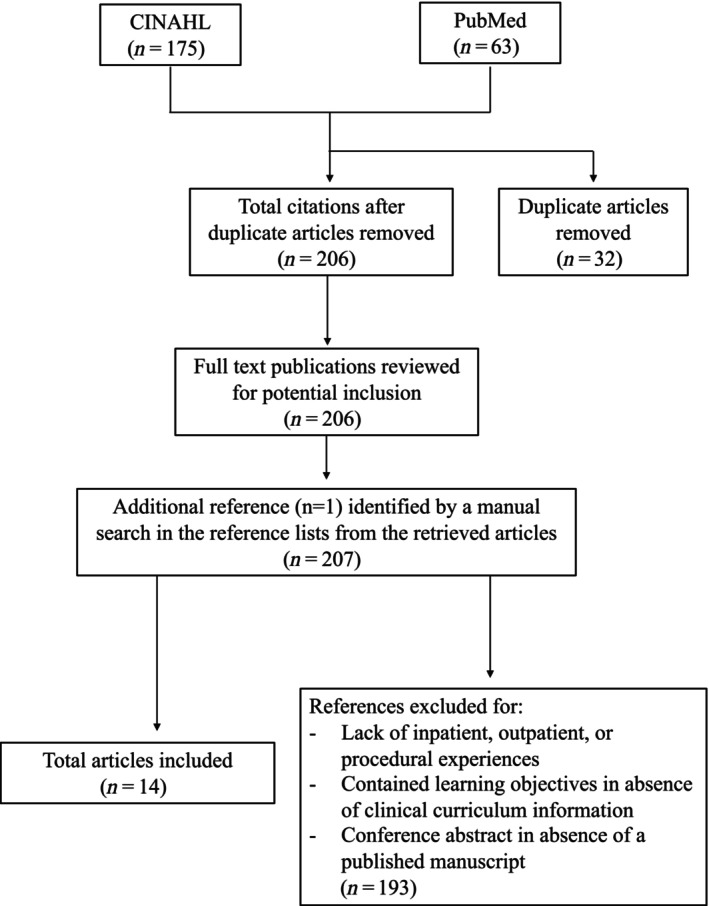
Flow chart of the literature search process for the present article. CINAHL, Cumulated Index to Nursing and Allied Health Literature.

The authors created inclusion and exclusion criteria to select articles for a narrative review. Articles that were included described formal student experiences in PM&R during preclinical or clinical years with the description of the rotation including inpatient, outpatient, or procedural physiatry. Articles that were excluded were those that focused on aspects of education outside of clerkships or rotations, such as workshops, student interest fairs, and student interest groups. Additionally, articles that included learning objectives in the absence of clinical curriculum information were also excluded. Lastly, abstracts in the absence of a published manuscript were excluded. In collaboration with a medical librarian, a search strategy was developed with combined terms for PM&R and clerkship rotations (keywords consisted of physical and rehabilitation medicine, rehabilitation, physiatry, clinical clerkship, medical students). The search in PubMed yielded 63 results, and a search in the Cumulated Index to Nursing and Allied Health Literature (CINAHL) database yielded 175 articles. After 32 duplicate citations were removed, a total of 206 articles were evaluated independently by two study authors. One additional article that fit the inclusion criteria was identified through review of references from selected articles. In total, 14 articles qualified for narrative review based on inclusion and exclusion criteria (Figure 1).

### General PM&R and combined PM&R/neurology clerkships

Since 2000, five papers have detailed the curriculum for required in‐person PM&R clerkships. Clerkships were 2 to 4 weeks in duration and typically required time spent in both the inpatient and outpatient settings. The specific design elements, educational interventions, and assessments for each rotation are outlined in Table 1.

**TABLE 1:** Comparison of neurorehabilitation PM&R clerkship designs since the year 2000.

Authors	Laskowski et al.^8^	Faulk et al.^9^	Norbury et al.^10^,^a^	Curtis et al.^11^	Hartsgrove et al.^12^
Level	Second year	Fourth year	Fourth year	Fourth year	Fourth year
Duration	3 weeks required	2 weeks required	2 weeks required	4 weeks required	2 weeks required
Design	Week 1: 4‐h (half day) lecture/workshop sessions on PM&R basics Weeks 2 & 3: four half day clinical rotations in outpatient and inpatient PM&R	General inpatient rehab ×1 week Subspecialty inpatient rehab ×1 week 2–3 or 4–5 half days in outpatient clinics	Inpatient (50%) Outpatient (50%)	Combined neurology/PM&R clerkship PM&R: inpatient, outpatient Neurology: neurological ICU, inpatient, outpatient, consults	Daily hands‐on clinical experiences
Didactic sessions		Case‐based discussions Readings from AAPM&R study guides Daily resident lectures	Readings from *Current Medical Diagnosis and Treatment (CMDT) 2015* Two 60 min physical exam workshops 2 h case based workshop 60 min interactive case‐based lecture 60 min “chalk talk” Grand rounds Resident lectures	Nine didactic presentations Four laboratory sessions	8 h total Lectures on general physical disabilities Panel discussion with persons with disabilities
Assessments	Week 1: MSK skills acquisition test (103 items, given by physical therapist) Weeks 2 & 3: history and physical exam skills, written assignment on impairments and disability, final written examination (100 questions)	Neuro/MSK physical exam Oral presentation on PM&R topic Final written exam (30 multiple choice)	Final written exam	Three OSCEs Final written exam	
Grading scheme	Performance on clinical rotations Written examination Skills acquisition test	Clinical performance (60%) Professionalism (20%) Written final exam (20%)			
Additional resources	Physical exam videos Descriptions of the physical exam maneuvers Reference textbooks Detailed handouts	PM&R pocket reference book Online hub for reading materials and schedule		“The Purple Book” ‐ provided logistical and neurology/PM&R educational information	

Abbreviations: AAPM&R, American Academy of Physical Medicine and Rehabilitation; ICU, intensive care unit; MSK, musculoskeletal; OSCE, Objective Structured Clinical Examinations; PM&R, physical medicine and rehabilitation.

^a^ Revised curriculum based on Faulk et al.^9^

Laskowski et al. described the curriculum for a required PM&R clerkship in a well‐established PM&R program.^8^ In this curriculum, second‐year medical students participated in a 3‐week required clerkship. After the first week of PM&R lectures, the students' musculoskeletal skills were evaluated by physical therapists using a skills acquisition test. By 15–20 months after the examination, 82% of physical exam skills were retained.

In Faulk et al.,^9^ students spent 1 week on a general rehabilitation inpatient service and 1 week on a specialty inpatient unit (spinal cord injury [SCI], traumatic brain injury [TBI], or pediatric rehabilitation), with half days of outpatient clinic incorporated into the schedule. The authors found that self‐reported knowledge of PM&R showed statistically significant increases across 19 domains, and 73% of students found the rotation to be satisfactory.^9^

Due to lower quality assessments of the PM&R clerkship compared to other required clerkships in their medical school, Norbury et al.^10^ describes revisions that were made to the clerkship outlined in Faulk. These changes sought to achieve better alignment with the medical school's mission of training primary care physicians. As such, assigned reading was changed from American Academy of Physical Medicine and Rehabilitation (AAPM&R) study guides to *Current Medical Diagnosis and Treatment (CDMT) 2015*. Content was revised to focus more on general neurological and musculoskeletal pathologies and less on rehabilitation‐specific information. They also incorporated more didactic sessions with physical examination and case‐based workshops. The authors interpreted the results of their study to suggest that learning material should be presented in different formats and that students were more satisfied with content that was better tailored to a primary care perspective.^10^

Curtis et al.^11^ completed a unique study detailing an integrated neurology‐PM&R core clerkship and how this related to fourth‐year medical student skills and attitude. The clerkship consisted of both inpatient and outpatient experiences for both neurology and PM&R. Results of the study showed an improvement in Objective Structured Clinical Examinations (OSCEs) performance for the musculoskeletal and neurological examinations and self‐reported confidence domains in multiple different clinical abilities. Feedback from students suggested that the high variety in clinics negatively affected continuity and that they would have preferred the rotation to be more tailored to their chosen specialties.^11^

Most recently, a study by Hartsgrove et al.^12^ explored the benefit of a mandatory, 2‐week, fourth‐year rotation by performing a survey study. The clerkship contained daily, hands‐on clinical experiences, and 8 hours total of didactics pertaining to general physical disabilities, SCI, agitation after a brain injury, and interacting directly with people with disabilities to discuss the lived experience. The article did not outline the specifics of the hands‐on clinical experiences, whether they were inpatient or outpatient, nor the assessments used during the rotation. Notably, medical students reported increased perceived comfort and knowledge of treating people with disabilities and increased confidence in caring for people with disabilities, and 6 months following the rotation, 80% of students reported they were still integrating the knowledge they gained from their PM&R clerkship into their other clinical experiences.

Two articles published prior to 2000 described PM&R clerkship curricula. The mandatory 2‐week PM&R clerkship described in Kirshblum et al.'s report^13^ consisted of 20 hours of lectures and a written examination at the end of the rotation. Each student rotated with a resident and an attending physician, although it is unclear if this was in the inpatient or outpatient setting. Students were given a pre‐ and post‐clerkship questionnaire that assessed their basic knowledge of the PM&R field. Authors concluded that their PM&R clerkship improved medical students' overall awareness of physiatry.^13^

Katz et al.^14^ described a 1‐week PM&R clerkship in which students were exposed to inpatient PM&R. The rotation also included formal clinical case presentations and lectures with an emphasis on highlighting the role of each member of the rehabilitation team. Of note, this rotation was made possible by taking 1 week away from the students' neurology rotation. In open‐ended comments at the end of the rotation, students expressed that they approached the rotation with a negative attitude due to losing a week of their neurology rotation, but they ultimately found the rotation to be a positive and rewarding experience.

Over time, mandatory PM&R clerkships have evolved, and many themes have emerged from more recent studies and clerkships. One is a more multifaceted approach to teaching rehabilitation medicine with incorporation of increased hands‐on learning, using cadaver labs, OSCEs, case presentations, and discussions, in addition to classic lectures.^13^ Clerkships described in older articles in general had greater hours of lecture incorporated than those described in more recent articles. For example, Kirshblum et al.'s experience provided 20 hours of didactics and Norbury et al. reported a maximum of 12.5 hours of didactics.^10^, ^13^ The clinical aspect of more modern clerkships also appears more robust, with greater time spent in PM&R clerkships, typically 2 or 4 weeks, and therefore providing a richer experience in inpatient and outpatient rehabilitation practice settings. The main diagnoses seen have also changed over time. Katz et al.'s report^14^ focused primarily on SCI, TBI, amputees, and chronic pain, whereas more modern clerkships have also included cancer rehabilitation, medically complex rehabilitation, women's health rehabilitation, post‐COVID rehabilitation, and more.

### Outpatient musculoskeletal clerkships

Musculoskeletal and spine symptoms are highly prevalent in physician offices, with up to 33.9% of community‐dwelling adults experiencing back pain in the last 3 months.^15^ Experience with the musculoskeletal system, including anatomy, kinesiology, and pathophysiology, is a core component of physiatric training and practice.^16^ As such, medical students who rotate in PM&R will gain exposure to patients with musculoskeletal conditions and the opportunity to complete a comprehensive evaluation. A study by Altschuler et al. reviewed the experience of fourth year medical students enrolled in a mandatory 2‐week PM&R clerkship and used a checklist to evaluate learning of physical examination maneuvers.^17^ Twenty physical examination maneuvers were assessed prerotation and postrotation for correctly performing the examination, as determined by a PM&R attending physician, fellow, or resident. The study found that prerotation, 35% of exam maneuvers were completed correctly, whereas 82% were performed correctly postrotation, with improvements in 19 out of 20 maneuvers. The authors concluded that use of the checklist was helpful in teaching the musculoskeletal examination and that learning the musculoskeletal examination can be helpful for future practice in any specialty.

### Virtual clerkships

During the peak of the COVID‐19 pandemic, when medical students were unable to physically attend clerkships, virtual clerkships became an option for students seeking clinical exposure in certain specialties. Many PM&R residency programs turned to these virtual options to allow students to gain exposure to the field while maintaining social distancing.

Zeldin et al. altered their 4‐week in‐person neurology‐PM&R rotation to include 2 weeks of remote learning and 2 weeks of in‐person inpatient and outpatient exposure.^18^ At the end of the rotation, OSCE scores for neurology and PM&R standardized patients were consistent with prepandemic performances. The authors concluded that the hybrid in‐person/virtual curriculum can be an effective method for teaching foundational skills such as the physical examination.

Two other studies described virtual PM&R clerkships involving 2‐week rotations. The rotation in the article published by Farr et al.^19^ involved students participating virtually in inpatient rounding, outpatient clinics, obtaining medical history, and multidisciplinary team rounds. Students also attended virtual resident didactics and specific medical student didactics. The other published rotation was outlined in the article by Huang et al.^20^ Students were brought along on a tablet for 1 week of inpatient (rounds, multidisciplinary team rounds) and 1 week of outpatient (video‐assisted virtual history taking, physical exam instruction and observation, discussion of differential diagnosis, observed electrodiagnostic and ultrasound procedures). Students also attended resident didactics, grand rounds, and Morbidity & Mortality conference, and gave a 10‐minute virtual presentation.

Overall, these virtual rotations attempted to incorporate students in many aspects of PM&R, including inpatient, outpatient, and procedural work. However, the authors noted that these clerkships fell short in certain domains in which physical presence is necessary, such as practicing physical examination or procedural skills and assessing a student's interprofessional skills. Ultimately, these studies drew similar conclusions: although a virtual clerkship can provide education to students when in‐person options are not available, virtual clerkships remain inferior to traditional clerkships.

### PM&R externships/early clinical experiences

With PM&R being a smaller, lesser‐known medical specialty, early exposure to the field during medical school is one way to increase the number of students entering the field and to provide students an early understanding of the field of PM&R.

The Medical Student Summer Clinical Clerkship Externship is an 8‐week program sponsored by the Association of Academic Physiatrists.^21^ It is designed for students to participate during the summer between their first and second years of medical school. During the program, students participate in inpatient and outpatient rotations, resident didactic education, and online teleconferences. In Canada, a 2‐week program between the second and third year of medical school incorporated shadowing of physiatrists, participating in workshops (tours of hospital, observing history and physical, botulinum toxin and baclofen pump refills), lunchtime talks (1‐hour by attending physicians on topics such as residency training, subspecialties, and specific skill sets), and skills practice (joint injections on mannequins).^22^

The objectives of these early clinical exposure programs were less aimed at learning the medical management of specific disease states within the field of PM&R but instead were targeted at understanding the role of physiatry in an interdisciplinary team, the breadth of the field, and broad social/medical issues surrounding patients with disabilities. These early exposure experiences are imperative for spreading awareness of the field for preclinical medical students and providing education on how to care for individuals with disabilities.

## DISCUSSION

Based on this review, PM&R clerkships and externships can be valuable in undergraduate medical education to gain experience working with patients with musculoskeletal, neurologic, or other disabling conditions. These clinical PM&R experiences can also be beneficial for career exploration. Overall, there is limited information available regarding the most effective methods or settings to teach PM&R clinical skills to medical students. Based on this review, PM&R clerkships currently involve a wide variety of educational methods, including inpatient and outpatient clinical experiences, lectures, OSCEs, case discussions, written examinations, physical examination sessions, cadaver sessions, small group discussion, and virtual education. Since the 1980s, clerkships have evolved to incorporate greater use of technology, hands‐on learning techniques, and more time in unique subspecialties of the field of PM&R. Based on different assessments, in various PM&R clerkships, medical students demonstrated improved musculoskeletal and neuromuscular physical examination skills and increased knowledge and confidence in PM&R‐related clinical knowledge.

Medical students may choose to participate in an elective PM&R rotation for career exploration purposes, and these clerkships allow students who are considering PM&R as a medical specialty to experience hands‐on, direct clinical experiences in inpatient, outpatient rehabilitation, or procedural settings. For those students participating in a PM&R clerkship who are *not* specifically considering PM&R as a career, clerkships can still allow for important educational opportunities based on this review. These clerkships can serve as a meaningful way for students to improve their neurologic and musculoskeletal physical examination skills and PM&R knowledge.

Early clinical opportunities during preclinical years are excellent opportunities for medical students to experience the field of PM&R in inpatient, outpatient, and procedural settings. These programs are crucial for exposing medical students to the field, especially those who do not have a PM&R department at their home medical school. Additionally, virtual clerkships can achieve similar aims by exposing medical students in their clinical years to the field of PM&R. However, these virtual clerkships fall short in areas in which physical presence is necessary and should be reserved for when in‐person rotations are not feasible.

Ideally, future curriculum development in PM&R clinical settings can use the medical literature to incorporate proven strategies. More research is needed to understand the optimal PM&R clinical skills to teach in PM&R clerkships, as well as the most effective ways to teach PM&R clinical skills, which specific teaching techniques (large lectures, small group discussions, hands‐on labs, etc.) are most effective for fulfilling a program's aims, and which educational opportunities are most beneficial for students to achieve medical school competencies or career exploration goals.

## CONCLUSION

This review summarizes the available literature on PM&R clinical experiences in undergraduate medical education. PM&R clerkships have been shown to help medical students learn more about the field of PM&R and improve musculoskeletal and neuromuscular physical examination skills, as well as acquire PM&R specific knowledge. Further research is necessary to determine the optimal clinical curriculum for PM&R including which clinical skills to target and which methods are most effective, in order to shape future PM&R curriculum development and educational innovations.

## DISCLOSURE

The authors have no competing interests for financial benefits.
